# Accuracy of genomic prediction for milk production traits in Mehsana buffalo

**DOI:** 10.3389/fgene.2026.1839396

**Published:** 2026-06-02

**Authors:** Mayank R. Patel, Ashish C. Patel, Rajesh S. Joshi, Devanshi V. Patel, Nilesh Nayee, A. Sudhakar, Subhash J. Jakhesara, Prakash G. Koringa

**Affiliations:** 1 Department of Animal Genetics and Breeding, College of Veterinary Science and Animal Husbandry, Kamdhenu University, Anand, Gujarat, India; 2 National Dairy Development Board, Anand, Gujarat, India; 3 Department of Veterinary Biotechnology, College of Veterinary Science and Animal Husbandry, Kamdhenu University, Anand, Gujarat, India

**Keywords:** genomic prediction, Mehsana buffalo, PBLUP, prediction bias, ssGBLUP

## Abstract

**Introduction:**

Genomic information can contribute significantly to the increase in the accuracy of genetic evaluation compared to relying solely on pedigree relationships. Hence, the objective of this study was to compare the accuracy of genomic prediction for 305 days milk yield (305DMY), 305 days fat yield (305DFY), 305 days solid-not-fat yield (305DSNFY) and 305 days protein yield (305DPY) traits in Mehsana buffalo using pedigree best linear unbiased prediction (PBLUP) and single-step genomic best linear unbiased prediction (ssGBLUP) models. Prediction bias was assessed by estimating the regression coefficient of corrected yield (Yc) on predicted breeding values (BVs).

**Methods:**

The phenotype dataset comprised of test day records of 10,897 Mehsana buffalo for milk yield, 10,896 for fat yield, 10,581 for SNF yield and 10,578 for protein yield were used for present study. A total of 4,107 blood samples of Mehsana buffaloes were collected and genotyped using BUFFCHIP 54K SNP array. After the quality control, final dataset comprised of 3,887 Mehsana buffalo with 53,292 SNPs.

**Results:**

The prediction accuracies obtained using PBLUP ranged from 0.078 to 0.088, whereas those from ssGBLUP ranged from 0.088 to 0.100. The average predictive accuracies across traits were 0.083 for PBLUP and 0.093 for ssGBLUP, representing an overall improvement of 11.37% with ssGBLUP. The regression coefficients of predictions ranged from 0.44 to 0.52 for PBLUP and from 0.45 to 0.61 for ssGBLUP across production traits. The average regression coefficients were 0.48 for PBLUP and 0.53 for ssGBLUP, indicating reduced prediction bias under the ssGBLUP model.

**Conclusion:**

ssGBLUP provides more accurate and less biased breeding value predictions than PBLUP for production traits in Mehsana buffaloes.

## 1 Introduction

Livestock rearing plays a vital role in the economy of many countries, particularly in rural areas, by providing income and contributing to the national food supply. In India, the livestock sector employs approximately 8.8% of the population, contributing to 5.5% of the overall gross value added (GVA) and 31% of the agricultural GVA. Among livestock species, buffaloes hold significant economic and cultural importance, especially in developing countries like India, owing to their high potential for milk production, meat yield, and use as draught animals.

India has a substantial buffalo population, with Mehsana buffaloes recognized as one of the important dairy breeds due to their persistent milk production and regular breeding performance. However, despite the significant contribution of buffaloes to milk production in India, the genetic improvement of these animals has been slow. Genetic improvement is directly proportional to the intensity of selection, accuracy of selection and additive genetic standard deviation of traits while inversely proportional to the generation interval (Falconer, 1996).

Traditional breeding methods based on phenotypic performance and pedigree information have limited efficiency, particularly in identifying superior breeding animals at an early age. Although progeny testing programs provide higher accuracy, they are time-consuming and costly to implement (Bouquet and Juga, 2013). Recent advances in molecular genetics have introduced tools such as quantitative trait loci (QTL) mapping and marker-assisted selection (MAS) to enhance genetic improvement; however, these approaches account for only a small proportion of the total genetic variance (Yadav et al., 2018). On the other hand, genomic selection offers a more comprehensive approach by considering the effects of all markers throughout the genome. Genomic selection has proven effective in accelerating genetic progress in dairy animals and holds considerable potential to revolutionize breeding programs by reducing generation intervals (Meuwissen et al., 2016). The accuracy of breeding values (BVs) is crucial for the success of genomic selection. It depends on factors such as the predictive models used, the size of the reference population, heritability of traits and the extent of linkage disequilibrium between markers and QTL (Larkin et al., 2019).

Currently, improving the accuracy of genomic breeding values has been recognized as a crucial step for genomic improvement. However, until now, no studies have evaluated the accuracies of genomic prediction for production traits using PBLUP and ssGBLUP models in Mehsana buffalo. Therefore, the objective of the present study was to determine the prediction accuracy and bias of genomic prediction models for 305 days milk yield (305DMY), 305 days fat yield (305DFY), 305 days SNF yield (305DSNFY) and 305 days protein yield (305DPY) traits from Mehsana buffaloes using PBLUP and ssGBLUP models.

## 2 Materials and methods

### 2.1 Phenotype data

In this study, monthly test-day records of morning and evening milk yields of Mehsana buffaloes, collected between 2008 and 2022, were retrieved from the INAPH (Information Network for Animal Productivity and Health) database (Nayee et al., 2016). The dataset included test-day milk yield (TDMY), test-day fat yield (TDFY), test-day solid-not-fat yield (TDSNFY) and test-day protein yield (TDPY) and is summarized in Table 1. For inclusion in the analysis, each animal was required to have at least three test-day records.

**TABLE 1:** Descriptive statistics of data to be used for PBLUP and ssGBLUP analyses.

Trait	No. of buffalo	No. of test day records	No. genotyped	Mean (kg)	SE	SD
TDMY	10,897	102,583	3,887	6.50	0.0070	2.2536
TDFY	10,896	101,538	3,838	0.46	0.0005	0.1659
TDSNFY	10,581	86,416	2,860	0.63	0.0007	0.2231
TDPY	10,578	86,558	2,842	0.25	0.0003	0.0963

### 2.2 Genotype data

A total of 4,107 blood samples of Mehsana buffaloes were collected under the project “Establishing Genomic Selection Network for Dairy Cattle and Buffalo Breeds in Gujarat.” Genomic DNA was extracted using the QIAcube HT system following the manufacturer’s protocol. The DNA samples were genotyped using the BUFFCHIP 54K SNP genotyping array developed by NDDB. The generated.CEL files were processed in Axiom Analysis Suite v5.2 with default quality control settings. Subsequently, raw genotype data were subjected to quality control in PLINK 1.9 (Chang et al., 2015). SNPs with >10% missing genotypes, minor allele frequency (MAF) < 0.05, or significant deviation from Hardy–Weinberg equilibrium (p < 1 × 10^−6^) were removed. Individuals with SNP call rates <90%, genomic relationships between animal >0.9, or extreme heterozygosity indicative of potential contamination were also excluded. Following quality control, the final dataset comprised 3,887 buffalo and 53,292 SNPs, with an overall genotyping rate of 0.99. No imputation was performed on residual missing genotypes, and all subsequent analyses were carried out on this filtered dataset.

Of the 3,887 genotyped buffaloes retained after quality control, the number with available matching phenotypic records varied across traits, as milk composition traits are not recorded with the same frequency as milk yield under the INAPH recording protocol. Specifically, 3,887 animals had test-day milk yield (TDMY) records, 3,838 had test-day fat yield (TDFY) records, 2,860 had test-day SNF yield (TDSNFY) records, and 2,842 had test-day protein yield (TDPY) records.

### 2.3 Statistical analysis

The BVs were estimated using the PBLUP and ssGBLUP model. The two models were described below.

ythijklmno =Hj+Lm+YCn+SCo+Oh+HYMRi+∑k=0nf∅ktlβl+∑k=0nr∅ktlukl+∑k=0nr∅ktl pekl+ethijklmno

Where, *y*_*thijklmno*_ is a production trait of *k*th animal made on *t*th days in milk (DIM); *H*_*j*_ is a herd as a fixed effect with subclass *j,* where herd refers to the village in which the animal was located (18 levels)*; L*_*m*_ is a lactation number as a fixed effect with subclass *m* (level 3)*; YC*_*n*_ is a year of calving as a fixed effect with subclass *n* (level 15*)*; *SC*_*o*_ is a season of calving as a fixed effect with subclass *o* (Level 3); *O*_*h*_ is an owner as a random effect with subclass *h; HYMR*_*i*_ is a herd x year of milk recording x month of milk recording as a random effect with subclass *I;* $\beta_{l}$ is a fixed regression coefficients; $u_{kl}$ and $pe_{kl}$ are the *l* ^th^ random regression for animal additive genetic and permanent environmental effects, respectively, for animal *k;* ∅ _*ktl*_ is the *l^th^* legendre polynomial for the test day record of animal *k* made on *t* ^*th*^ day in milk; *nf* is the order of polynomials for DIM; *nr* is the order of polynomials for animal and *pe* effects; and *e*_*thijklmno*_ is a random residual effect.

Variance components obtained by AIREML using pedigree information were used for both PBLUP and ssGBLUP breeding value estimation using DMU V 5.2 (Madsen et al., 2006). The variance components and breeding values obtained under random regression model were represented in terms of 305 days BVs using the Legendre polynomial covariates as per the procedure described by Mrode (2005) (Table 2).

**TABLE 2:** Variance components and heritability estimation using PBLUP model in Mehsana buffalo.

Trait	σ^2^P	σ^2^a	σ^2^other random	σ^2^e	h^2^
305DMY	187,733.744	58,788.56	102,297.796	26,647.39	0.31
305DFY	1,110.010	248.2174	566.338	295.4542	0.22
305DSNFY	1832.533	549.9156	990.301	292.3167	0.30
305DPY	344.787	85.11269	172.295	87.37858	0.24

σ^2^_P_ = Phenotypic variance, σ^2^_a_ = Additive genetic variance, σ^2^_e_ = Residual variance.

σ^2^_other random_ = Variance for other random effects of HYMR, and Owner, h^2^ = Heritability.

The primary distinction between the two methods lies in the relationship matrix used for genetic evaluation. The PBLUP approach estimates breeding values using the pedigree-based numerator relationship matrix (A-matrix). In contrast, the ssGBLUP approach integrates pedigree and genomic information by combining the A-matrix with the genomic relationship matrix (G-matrix) to construct the H-matrix. The inverse of the combined relationship matrix (H^−1^) was calculated according to Aguilar et al. (2010).

$$H^{-1}=A^{-1}+\left[\begin{array}{cc} 0 & 0\\ 0 & G^{-1}-A_{22}^{-1} \end{array}\right]$$

Here, A^-1^ and H^−1^ are the inverse of the numerator and genomic relationship matrix, respectively. $A_{22}^{-1}$ is the inverse of the pedigree relationship matrix for genotype animal only. The G matrix was created with the following equation (VanRaden, 2008):

$$G=\frac{{\text{ww}}^{\prime }}{{2\;ƩP}_{j}\left(1-P_{j}\right)}$$

Where, w is a centralized genotype matrix with rows as individuals and columns as markers and P_*j*_ is a frequency of the second allele for the *j*th marker.

### 2.4 Reliability of BVs

The reliability of BVs quantifies the accuracy and precision of the BVs and indicates the level of confidence one can have in using them for selection decisions. According to Mrode (2005), reliability was calculated as:

$$R=1-\left(\text{PEV}/G\right)$$

Where, R is the reliability; PEV is the predicted error variance for breeding value estimate; and G is the genetic variance for the trait.

### 2.5 Accuracy of prediction

The accuracy of prediction was assessed by using the five-fold cross-validation method (Luan et al., 2009). The basic idea of cross-validation was to randomly divide the reference population into five folds of approximately equal size. One fold was used for validation and the remaining four folds were used for training the model. The phenotypic records of animals in the validation data were masked and their breeding values were predicted from the information of training data. For each validation set, prediction accuracy was calculated as the correlation of corrected yield (Yc) of animals with respective BVs obtained in validation sets. The Yc of 305 days was calculated by removing fixed effect and non-additive random effects estimates from the observation of an animals. The correlation coefficients were computed only for those animals whose records were excluded in that particular validation set. In addition, BVs obtained in each validation set for the validation animal were pooled to determine the overall correlation coefficient and compare the prediction ability of both the models.

### 2.6 Prediction bias

To assess the bias of prediction, the regression coefficient of corrected yield (Yc) on BVs was calculated. The slope of the regression line indicated the presence of bias in the genomic predictions. A slope less than or greater than one indicates an overestimation or underestimation, respectively.

## 3 Results

The variance components and heritability estimates for the milk production traits are presented in Table 2. The range of heritability for the four traits was between 0.22 and 0.31. Among all investigated traits, the estimated heritability of 305DMY was the highest (0.31) and the lowest was for 305DFY (0.22).

### 3.1 Reliability of BVs

The reliability of breeding values for production traits estimated using the PBLUP and ssGBLUP models ranged from moderate to high (Table 3). Across all traits, ssGBLUP yielded higher reliability estimates compared to PBLUP. The improvement in reliability achieved through ssGBLUP ranged from 1.13% to 1.61% for production traits, demonstrating the advantage of incorporating genomic information into the evaluation process.

**TABLE 3:** Comparison of breeding value reliabilities estimated by PBLUP and ssGBLUP models.

Trait	PBLUP	ssGBLUP	% reliabilities increased
305DMY	0.636	0.644	1.26
305DFY	0.567	0.576	1.59
305DSNFY	0.622	0.629	1.13
305DPY	0.558	0.567	1.61

### 3.2 Accuracy of prediction

The prediction abilities of the PBLUP and ssGBLUP models for production traits, evaluated using five-fold cross-validation are presented in Table 4. The accuracy of prediction obtained using PBLUP ranged from 0.078 to 0.088, whereas those from ssGBLUP ranged from 0.088 to 0.100. The average predictive accuracy across traits were 0.083 for PBLUP and 0.093 for ssGBLUP. This reflects an overall improvement of 11.37% when using ssGBLUP.

**TABLE 4:** Accuracy of prediction for PBLUP and ssGBLUP models.

Traits	PBLUP	ssGBLUP	% Predictability increased
305DMY	0.083	0.088	6.02
305DFY	0.085	0.089	4.70
305DSNFY	0.088	0.100	13.64
305DPY	0.078	0.095	21.80

### 3.3 Prediction bias

**FIGURE 1:**
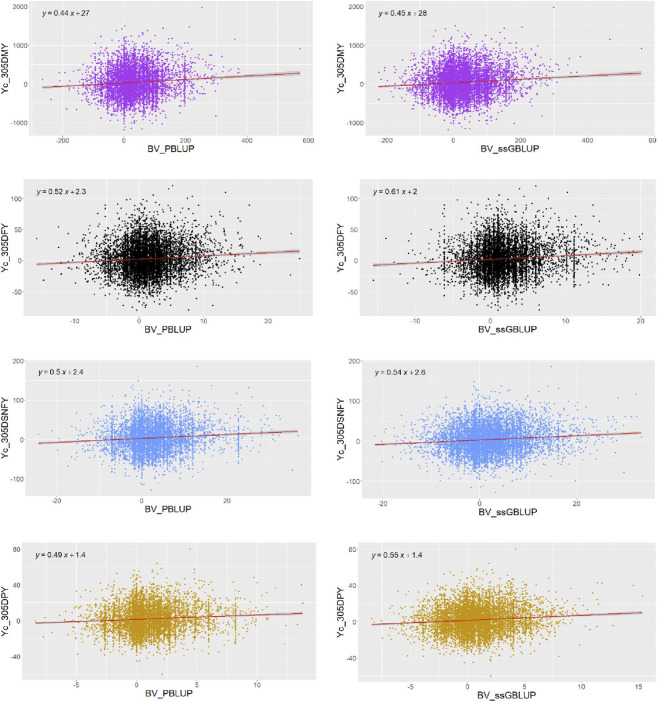
Regression coefficient of the corrected yield of BVs for PBLUP and ssGBLUP model.

The use of regression coefficients is a common practice in animal breeding to identify factors that influence the accuracy of genomic predictions and adjust for bias to improve the estimates. In the present study, the regression coefficients of predictions obtained using the PBLUP and ssGBLUP methods ranged from 0.44 to 0.52 and 0.45 to 0.61, respectively, across all production traits (Figure 1). For both models, the slope of the regression line was less than one, indicating an overestimation of breeding values. The average regression coefficients across traits were 0.48 for PBLUP and 0.53 for ssGBLUP.

## 4 Discussion

The heritability estimates for production traits in Mehsana buffalo indicated moderately heritable, ranging from 0.22 to 0.31 (Table 2). The heritability estimate for 305DMY was 0.31, which was close to the estimate of 0.32 reported by Sharma et al. (2023) in Murrah buffalo, but higher than the estimates of 0.22, 0.24, and 0.18 reported by Aspilcueta-Borquis et al. (2010), Yousuf et al. (2017) and Singh et al. (2015) respectively, in Murrah buffalo, as well as the estimate of 0.28 reported by Sathwara et al. (2020) in Mehsana buffalo. The heritability estimate for 305DFY was 0.22, which was similar to the estimate of 0.21 reported by Aspilcueta-Borquis et al. (2010) in Murrah buffalo, but lower than the estimates of 0.28 and 0.33 reported by Sathwara et al. (2020) in Mehsana buffalo and Kumar et al. (2016) in Murrah buffalo, respectively, and higher than the estimate of 0.14 reported by Flores et al. (2013) in Philippine dairy buffaloes. The heritability estimate for 305DPY was 0.24, which was similar to the estimate of 0.23 reported by Aspilcueta-Borquis et al. (2010) in Murrah buffalo, but higher than the estimate of 0.19 reported by Flores et al. (2013) in Philippine dairy buffaloes. The heritability estimate for 305DSNFY was 0.30, which closely aligned with the estimate reported by Chitra et al. (2016) in Murrah buffalo.

The reliability of breeding value serves as a critical measure in assessing the accuracy of predictions related to an individual’s genetic merit for specific traits. The reliability of BVs estimated using ssGBLUP compared to PBLUP increasing the 1.13%–1.61%. This increase in reliability can be attributed to the incorporation of genotype animal information into the ssGBLUP model. These findings align with the results reported by Zhang et al. (2022), who also observed higher reliability ratios in ssGBLUP compared to PBLUP, with an increase ranging from 1% to 1.8% for production traits.

In this study, we investigated the prediction ability of the breeding values for production traits using PBLUP and ssGBLUP models in Mehsana buffalo. Maximizing the profitability of the dairy industry requires a focus on selecting animals with high breeding values for economically important traits, particularly milk production traits (Wahinya et al., 2022). Breeding values are estimates of an animal’s genetic potential for passing on specific traits to its offspring. By identifying animals with superior breeding values for milk production traits, the dairy industry can accelerate genetic improvement and meet the growing demand for dairy products. Several previous studies (Nayee et al., 2018; Herrera et al., 2021; Zhang et al., 2022) have shown that the ssGBLUP outperformed PBLUP models for the prediction of breeding values in production traits.

Our result indicated that the prediction ability increased by 6.02% for 305DMY, 4.70% for 305DFY, 13.64% for 305DSNFY and 21.80% for 305DPY upon switching to the ssGBLUP from the PBLUP model. The average predictive accuracy across traits were 0.083 for PBLUP and 0.093 for ssGBLUP, reflecting an overall improvement of 11.37% with the ssGBLUP model. This improvement can be attributed to the simultaneous use of pedigree, phenotypic, and genomic information in the ssGBLUP model, which provides additional information for breeding values estimation compared to traditional pedigree-based models that rely solely on capturing Mendelian sampling variation. However, the prediction accuracies found in the current study were generally low but consistent with the ranges reported in previous research using small numbers of genotypes in ssGBLUP for traits such as milk production traits. Herrera et al. (2021) observed higher genomic prediction accuracy using the ssGBLUP model compared to PBLUP for three production traits in Philippine dairy buffaloes, with an average improvement of 7%. Likewise, Nayee et al. (2018) reported a 1.96% increase in prediction accuracy for the 305DMY trait in Holstein crossbred cattle when using ssGBLUP instead of PBLUP. In another study, Haque et al. (2025) observed 24.5% higher prediction accuracy using the ssGBLUP model compared to PBLUP for 305DMY in first lactation data of Korean Holstein cow. Gómez-Carpio et al. (2026) reported a 3–12% higher prediction accuracy of ssGBLUP model over PBLUP for production traits in Italian Mediterranean buffalo. Despite ssGBLUP consistently outperforming PBLUP in the current study, the relatively low prediction accuracy in this study is due to the limited number of genotyped animals, the degree of relatedness among them, and the characteristics of the prediction models used (Zhang et al., 2019).

Beyond evaluating prediction accuracy, assessing prediction bias is equally important when comparing models or designing a breeding program. The regression coefficients for all traits obtained from the PBLUP and ssGBLUP models (Figure 1) were less than one, indicating overestimation of breeding values across traits. The average regression coefficients for production traits were 0.48 for PBLUP and 0.53 for ssGBLUP, reflecting a relatively lesser bias (closer to 1) under the ssGBLUP model. These findings are consistent with those of Herrera et al. (2021), who reported average regression coefficients of 0.54 for PBLUP and 0.84 for ssGBLUP in dairy buffaloes. Notably, the degree of overestimation was higher under the PBLUP model, whereas ssGBLUP exhibited comparatively reduced bias, further highlighting the advantage of incorporating genomic information in the evaluation process. In another study, Gao et al. (2018) observed regression coefficients closer to one for PBLUP than for ssGBLUP across production traits in Finnish red dairy cattle, indicating that the extent of prediction bias may vary depending on population structure, trait architecture, and the size and composition of the reference population.

The remaining bias observed under ssGBLUP is primarily attributable to the limited size of the genotyped reference population, as well as incompatibility between the G-matrix and the A-matrix arising from selective and partial genotyping (Christensen and Lund, 2010; Misztal et al., 2014). Addressing this bias will be important for the practical implementation of genomic selection in Mehsana buffalo. Expanding the reference population through systematic genotyping of additional animals is expected to substantially reduce overestimation, given that prediction accuracy and bias are both directly dependent on reference population size (Zhang et al., 2019). Furthermore, adjusted ssGBLUP models incorporating optimized blending, tuning, and scaling parameters during H^−1^ matrix construction have been shown to reduce prediction bias by 14%–17% compared to the standard ssGBLUP in populations with limited genotypes (Mafolo et al., 2026), and represent a promising avenue for improving the reliability of genomic evaluations in this breed.

Overall, our findings indicate that ssGBLUP generated a higher prediction accuracy and less prediction bias than the PBLUP models for production traits in Mehsana buffalo, which could be implemented in practical breeding programs.

## 5 Conclusion

This study determined the prediction accuracy and bias of breeding value estimation for production traits in Mehsana buffalo using PBLUP and ssGBLUP models. The results demonstrate that the ssGBLUP model provides a more accurate prediction and lesser bias than PBLUP model for all studied traits. It is worth noting that the ssGBLUP yielded on average 11.37% higher accuracy than PBLUP on production traits. Moreover, prediction bias of the ssGBLUP model is lesser than the PBLUP model. Therefore, the ssGBLUP can be considered as effectively improving the prediction accuracy of breeding value for Mehsana buffalo.

## Data Availability

The original contributions presented in the study are included in the article/Supplementary Material, further inquiries can be directed to the corresponding author/s.
