# Supplementary material for: Accuracy of genomic prediction for milk production traits in Mehsana buffalo
Source: Front Genet. 2026 Jun 2;17:1839396. doi: 10.3389/fgene.2026.1839396 (PMC13268600; doi:10.3389/fgene.2026.1839396)
Supplement: Supplementary file 1 [file Table1.docx]

Supplementary Material

# Supplementary Tables

**Table 1: Mehsana buffalo samples summary based on the Axiom Analysis Suit software threshold parameter**

| **Sample Criteria** | **No. of Samples** |
| --- | --- |
| Number of input samples | 4107 |
| Samples passing DQC | 4107 |
| Samples passing QC CR | 4083 |
| Samples passing Plate QC | 4083 |
| Number of failing samples | 24 |
| Number of samples analysed | 4083 |
| Average QC CR for the passing samples: | 98.765% |
| DQC = Dish quality control, QC CR = Quality control call rate | |

**Table 2: Mehsana buffalo samples summary based on the PLINK 1.9 software threshold parameter**

| **Sample Criteria** | **No. of Samples** | **No. of SNPs** |
| --- | --- | --- |
| No. of Input samples and SNPs | 4083 | 54210 |
| SNP with >10% missing genotypes per SNP, MAF< 0.05 and deviation from HWE (p < 1×10^-6^) | - | 918 |
| Individual with SNP call rates < 90%, genomic relationships between animal > 0.9 and extreme heterozygosity | 196 | - |
| Final Genotype dataset | 3887 | 53292 |
| MAF =Minor allele frequency, HWE= Hardy-Weinberg equilibrium | | |
